# Effects of fully immersive virtual reality training on cognitive function in patients with mild cognitive impairment: a systematic review and meta-analysis

**DOI:** 10.3389/fnhum.2024.1467697

**Published:** 2024-12-06

**Authors:** Jing Yu, Jingru Song, Qin Shen

**Affiliations:** School of Nursing, Zhejiang Chinese Medical University, Hangzhou, China

**Keywords:** fully immersive virtual reality, mild cognitive impairment, cognitive function, executive function, memory, attention, meta analysis

## Abstract

**Background:**

Mild cognitive impairment (MCI) is a prodromal stage of dementia. There is no specific medication to slow the progression of MCI. Recent studies have confirmed the positive effects of virtual reality (VR). However, the results are inconsistent due to different types of VR interventions, small sample sizes, and the varying quality of the literature. This study aimed to assess the effects of fully immersive VR on cognitive function in MCI patients.

**Methods:**

A systematic review of published literature was conducted using PubMed, Cochrane Library, Embase, CINAHL, Web of Science, SinoMed, CNKI, Wanfang, and VIP Database. The search period was from inception through March 1, 2024. Eligible studies were randomized controlled trials evaluating the effects of fully immersive virtual reality training on cognitive function in MCI patients. Two investigators independently performed literature screening, data extraction, and quality assessment; a meta-analysis of the included literature was performed using RevMan 5.4. The Cochrane Risk of Bias tool was used to assess the methodological quality.

**Results:**

A total of 11 randomized controlled trials with 525 patients were included. The meta-analysis showed that fully immersive virtual reality training had significant effects on global cognitive function (MD = 2.34, 95% CI [0.55, 4.12], *p* = 0.01); (MD = 0.93, 95% CI [0.30, 1.56], *p* < 0.01), executive function (SMD = -0.60, 95% CI [−0.84, −0.35], *p* < 0.01), and attention (MD = 0.69, 95% CI [0.15, 1.23], *p* = 0.01). Still, the difference in memory (SMD = 0.27, 95% CI [−0.24, 0.78], *p* = 0.30) was not statistically significant. Subgroup analyses showed that executive function could be improved only when the intervention duration was ≥40 h. In contrast, excessive training (≥30 times) was counterproductive.

**Conclusion:**

Fully immersive virtual reality training improved cognitive functioning, executive functioning, and attention in MCI patients but was less effective in improving memory. Subgroup analysis suggests that fully immersive VR training must ensure sufficient intervention duration while avoiding frequent interventions.

**Systematic review registration:**

https://www.crd.york.ac.uk/PROSPERO/, PROSPERO (CRD42024498629).

## Introduction

Mild Cognitive Impairment (MCI) is a precursor stage of dementia, and its main symptoms include a decline in cognitive functions such as attention, memory, and executive function. Statistical data indicates that approximately 46% of individuals diagnosed with MCI will subsequently develop dementia within the following 3 years (Jia et al., 2020). Currently, there are no specific drugs that are effective in slowing the progression of MCI. Therefore, early screening of MCI patients and implementation of timely and effective non-pharmacological interventions is an effective way to slow down the progression to dementia (Petersen et al., 2018).

Virtual reality (VR) training is an emerging human-computer interaction technology targeted to create artificial interaction scenarios with multi-sensory, fun, and motivational elements. It can significantly overcome temporal and spatial limitations and is gradually becoming an emerging tool for cognitive training and rehabilitation of patients with MCI (Jang et al., 2023). According to the level of immersion, VR can be categorized into non-immersive, semi-immersive, and fully immersive. Among them, fully immersive VR technology is based on stereoscopic projection, 3D displays, motion capture, and other interactive devices, which are more capable of constructing a real interactive and fully engaged virtual environment than non-immersive or semi-immersive ones (Hsu et al., 2022). Currently, some studies have shown that virtual reality can improve the cognitive function of patients with MCI. However, the different types of VR interventions, the small sample size of the studies, and the variable quality of the literature led to inconsistent elaboration of the findings. It is not possible to accurately determine that virtual reality training is more effective than traditional rehabilitation techniques (Riaz et al., 2021). Furthermore, previous studies have concentrated on non-immersive and semi-immersive VR techniques, and existing meta-analyses have combined different VR intervention types. There is no evidence of the effectiveness of fully immersive and interactive features on cognitive functioning in patients with MCI.

The purpose of this systematic review is to identify and analyze randomized controlled trials of fully immersive virtual reality training on cognitive functioning in patients with MCI in order to assess the effectiveness of fully immersive virtual reality training and to discover the benefits of fully immersive VR with full immersion and interaction features on cognitive functioning in patients with MCI.

## Materials and methods

### Protocol registration

This systematic review has been registered on the PROSPERO (CRD42024498629).

### Data sources and search strategy

This study was conducted according to the guidelines of the Preferred Reporting Items for Systematic Reviews and Meta-Analyses (PRISMA). We conducted a comprehensive search for relevant articles from various academic databases. Articles were retrieved from PubMed, Cochrane Library, Embase, CINAHL, Web of Science, SinoMed, China National Knowledge Infrastructure, Wanfang, and VIP Database. The last retrieval date was March 1, 2024, and a combination of subject terms and free terms were used, while important references in the articles were traced back to obtain additional relevant literature. The keywords used were “mild cognitive impairment,” “mild cognitive decline,” “MCI,” “cognitive impairment,” “virtual reality,” “immersive virtual reality,” “virtual environment,” “virtual rehabilitation,” “virtual game,” “virtual reality training.” The Chinese database used the Chinese translation of the above keywords. Taking PubMed as an example, the specific search strategy is as follows.

#1 Search: “Cognitive Dysfunction”[Mesh]
#2 Search: (mild cognitive impairment [Title/Abstract]) OR (mild cognitive decline [Title/Abstract]) OR (MCI [Title/Abstract]) OR (cognitive impairment [Title/Abstract]) OR (cognitive dysfunction [Title/Abstract])
#3 Search: #1 OR #2
#4 Search: “Virtual Reality”[Mesh]
#5 Search: (virtual environment [Title/Abstract]) OR (Virtual Reality [Title/Abstract])) OR (immerse virtual reality [Title/Abstract]) OR (virtual rehabilitation [Title/Abstract])) OR (virtual game [Title/Abstract]) OR (virtual therapy [Title/Abstract])) OR (virtual treatment [Title/Abstract]) OR (VR [Title/Abstract])
#6 Search: #4 OR #5
#7 Search: #3 AND #6

### Inclusion and exclusion criteria

The eligibility criteria based on the PICOS (Participant, Intervention, Comparison, Outcome, Study design) framework are as follows. (1) Participant: Patients with mild cognitive impairment. Diagnostic criteria included Mini-mental State Examination (11–26 points) and Montreal Cognitive Assessment (<26 points). (2) Intervention: Studies used fully immersive VR training. (3) Comparison: The control group received no intervention or received traditional conventional therapy, non-immersive, and semi-immersive VR training. (4) Outcome: The primary outcome of this study was cognitive function, evaluated using the Mini-Mental State Examination (MMSE), Montreal Cognitive Assessment (MoCA), Trail-making Test A/B (TMT-A/B), Digital Span Test (DST), Stroop Color and Word Test (SCWT), Symbol Digit Modalities Test (SDMT), Animal Fluency Test (AFT), Language Testing in Asia (LTA); Digit Breadth Test (DBT). Secondary outcomes were daily living function, evaluated using the Instrumental Activities of Daily Living Scale (IADL). (5) Study design: randomized controlled trial. Exclusion criteria: (1) full text or original data is unavailable; (2) combining other interventions except the fully immersive virtual reality training; (3) conference papers; (4) case reports; (5) articles written in a language other than English or Chinese.

### Data extraction

Two researchers trained in evidence-based medicine independently screened the studies based on title, abstract, and full text, then extracted and cross-checked data. In case of disagreement, a third researcher intervened to reach a consensus. The study was based on the Cochrane Handbook 5.1.0 for original data extraction (Higgins et al., 2011), which consisted of three categories: literature characteristics, study participant characteristics, and intervention plan. Literature characteristics included first author, year of publication, and country. Study participant characteristics included age, sample size, and diagnostic criteria. The intervention plan includes test and control group interventions, platforms and tasks for fully immersive virtual reality training, intervention frequency, intervention period, intervention duration, outcome indicators, and measurement tools.

### Quality and risk of bias assessments

Two researchers independently assessed the risk of bias in the included literature according to the risk bias assessment tool recommended in the Cochrane Handbook for Systematic Reviews. If there was a disagreement, a third researcher was asked to make a joint decision. The assessment included seven items: (1) generation of randomized sequences, (2) allocation concealment, (3) blinding of subjects and investigators, (4) blinding of outcome assessors, (5) completeness of results and loss of visits, (6) selective reporting, (6) other biases (baseline imbalance, conflict of interest). The evaluation results included “low risk of bias,” “high risk of bias,” and “unclear.” “Low” indicates that the risk of bias is low, “high” indicates that the risk of bias is high, and “unclear” indicates that the literature does not provide sufficient information for bias analysis.

### Data synthesis and analysis

We used the statistical software RevMan 5.4 to analyze the extracted scale data in combination with effect sizes. Considering that the outcome indicators were continuous data, mean difference (MD) or standardized mean difference (SMD) was used as the effect statistic. When the measurement methods and units are the same, MD is selected; otherwise, SMD is selected. Heterogeneity was judged by *χ^2^* test and *I^2^* statistic; if *p* > 0.1, *I^2^* ≤ 50%, a fixed effect model was chosen; if *p* ≤ 0.1, *I^2^* > 50%, the source of heterogeneity was judged by subgroup analysis and sensitivity analysis, if still not able to exclude apparent clinical heterogeneity, a random effect model was chosen. This study calculated a 95% confidence interval (95% CI), and *p* < 0.05 was considered statistically significant. In addition, if the number of studies was ≥4, funnel plot analysis was used to detect publication bias.

## Results

### Description of included studies

**Figure 1:**
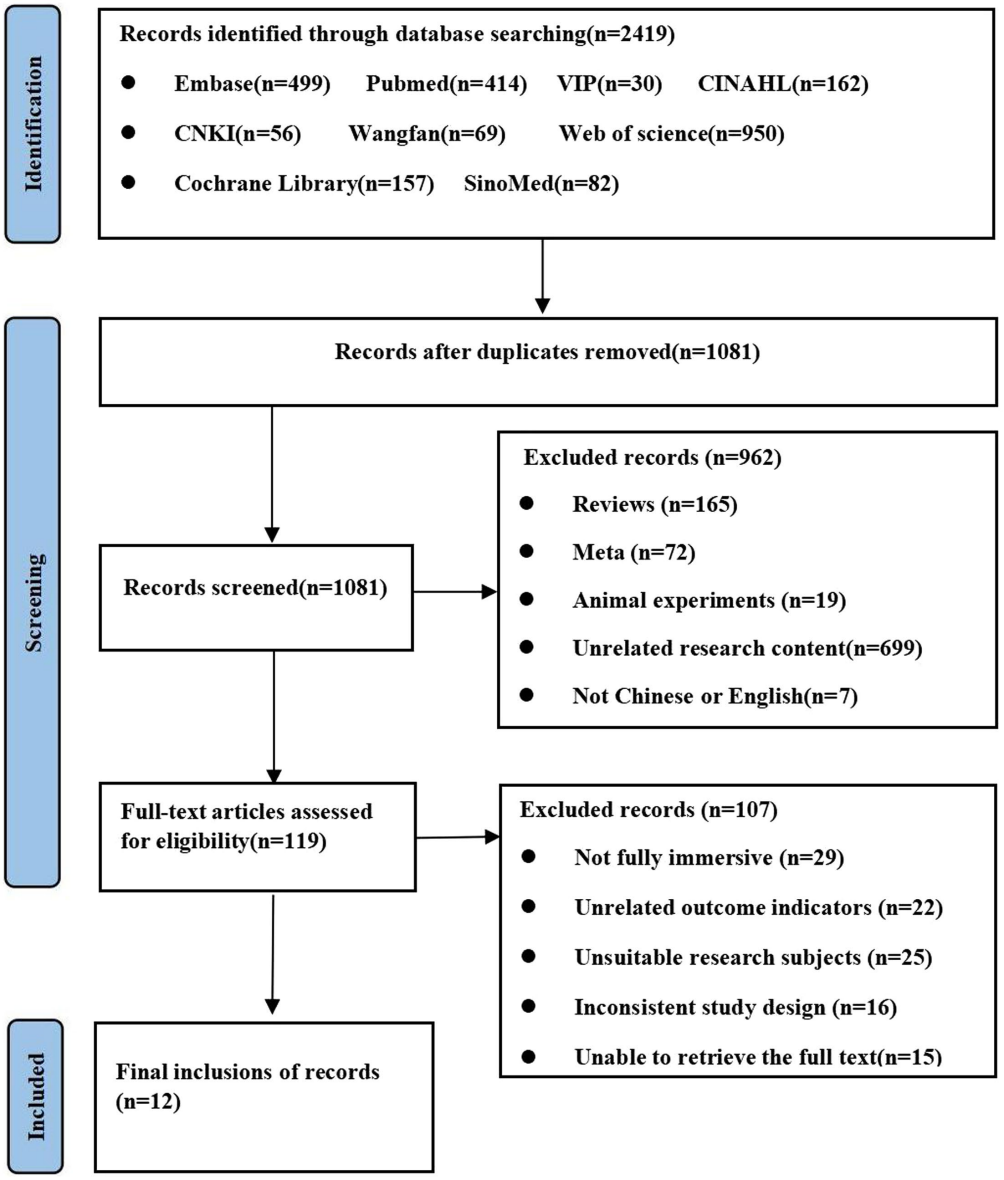
Literature screening process and results from each step of the process.

The included 12 studies, consisting of 11 RCTs, involved a total of 525 participants. The specific selection process is shown in Figure 1. Among the studies, there was a considerable difference in sample size, ranging from 21 to 66. The total duration of the training was from 12 to 60 h, and the total number of training sessions ranged from 20 to 72. More detailed information on the characteristics of the included studies is presented in Table 1.

**Table 1:** Characteristics of included studies.

Studies	Participate		Intervention		Frequency	Duration	Outcome
	Mean age (Mean ± SD)	Sample size (IG/CG)	IG	CG			
Liao et al. (2019)	75.5 ± 5.2/73.1 ± 6.8	18/16	Take the MRT, kitchen chef, convenience store clerk, Tai Chi, football (running and stepping)	traditional cognitive and physical training	60 min/times, three times/week	12 weeks	③④⑤
Park et al. (2020)	71.80 ± 6.61/69.45 ± 7.45	10/11	Crows and seagulls, automated Teller Machine, fireworks Party, shopping in the mart, fruit Cocktail	blank control	30 min/session, two times/week	12 weeks	①⑤⑨
Liao et al. (2020)	75.5 ± 5.2/73.1 ± 6.8	18/16	Taking the MRT, tai chi, looking for a store, stepping and running, kitchen chef, convenience store clerk	traditional cognitive and physical training	60 min/session, three times/week	12 weeks	②⑧⑩
Optale et al. (2010)	78.5 ± 10.9/81.9 ± 5.0	15/16	Noticing the path of a seagull’s flight view, leading the way to a destination, listening to training	musical training	Initial training: 30 min/repetition, three times/week. Intensive training:30 min/ session, two times/week.	12 weeks	①③④⑨⑩
Thapa et al. (2020)	72.6 ± 5.4/72.7 ± 5.6	33/33	Making juice, shooting crows, finding fireworks, correctly placing items	general health promotion	100 min/times, three times/week	8 weeks	①③④⑥
Baldimtsi et al. (2023)	66.07 ± 10.04/74.36 ± 7.04	28/28	Complete 20 simple numerical calculations and memorize the animals in the forest while riding your bike.	blank control	20 min/first 5 sessions, follow-up 30 min/session, 2–3 sessions/week	12 weeks	①④
Park (2022)	71.93 ± 3.11/72.04 ± 2.42	28/28	Program developed based on Unity game engine to perform the task of finding gems	traditional education and training	45 min/session, three times/week	8 weeks	⑧
Yang et al. (2022)	72.5 ± 5/72.6 ± 5.6	33/33	Making juice, shooting crows, finding fireworks, correctly placing items	health Seminar	100 min/times, three times/week	8 weeks	①③④⑥
Sun et al. (2024)	77.18 ± 6.41/78.03 ± 5.95	32/31	Ba Duan Jin training, supermarket shopping, wing flying, magic tricks, gym, space gravity ball	routine nursing services and health promotion	45 min/times, three times/week	24 weeks	②③④⑦⑨
Wang and Lu (2016)	59.61 ± 8.73	30/30	3D Differential Time Ranging Motion Capture Instrument Captures Patient’s 3D Motion Trajectory for Human-Computer Interaction to Complete ADL Training	routine acupuncture points	leave the needle for 40 min/times, during which the needle is rowed once every 10 min.		①②
Luo et al. (2022)	71.31 ± 6.74/74.19 ± 4.69	16/16	Fruit cutting task, obstacle course running task, picture matching task, jigsaw puzzle task, sorting training	routine rehabilitation training	30 min/session, five sessions/week	4 weeks	②③④⑨
Li (2023)	63.61 ± 2.33/65.40 ± 3.15	20/20	Basketball, obstacle track, picture matching, ATM withdrawal, trash sorting, object naming, maze game	routine cognitive training	30 min/session, five sessions/week	4 weeks	②

The outcome indicators were measured with the following instruments: Overall cognitive function ① Mini-Mental State Examination (MMSE); ② Montreal Cognitive Assessment (MoCA). Executive Function: ③ Trail-Making Test A (TMT-A); ④ Trail-Making Test B (TMT-B); ⑤ Stroop Color and Word Test (SCWT); ⑥ Symbol Digit Modalities Test (SDMT). Memory: ⑦ Animal Fluency Test (AFT); ⑧ Language Testing in Asia (LTA). Attention: ⑨ Digit Breadth Test (DBT). Daily living ability: ⑩ Instrumental Activities of Daily Living Scale (IADL).

### Literature quality and publication bias risk

**Figure 2:**
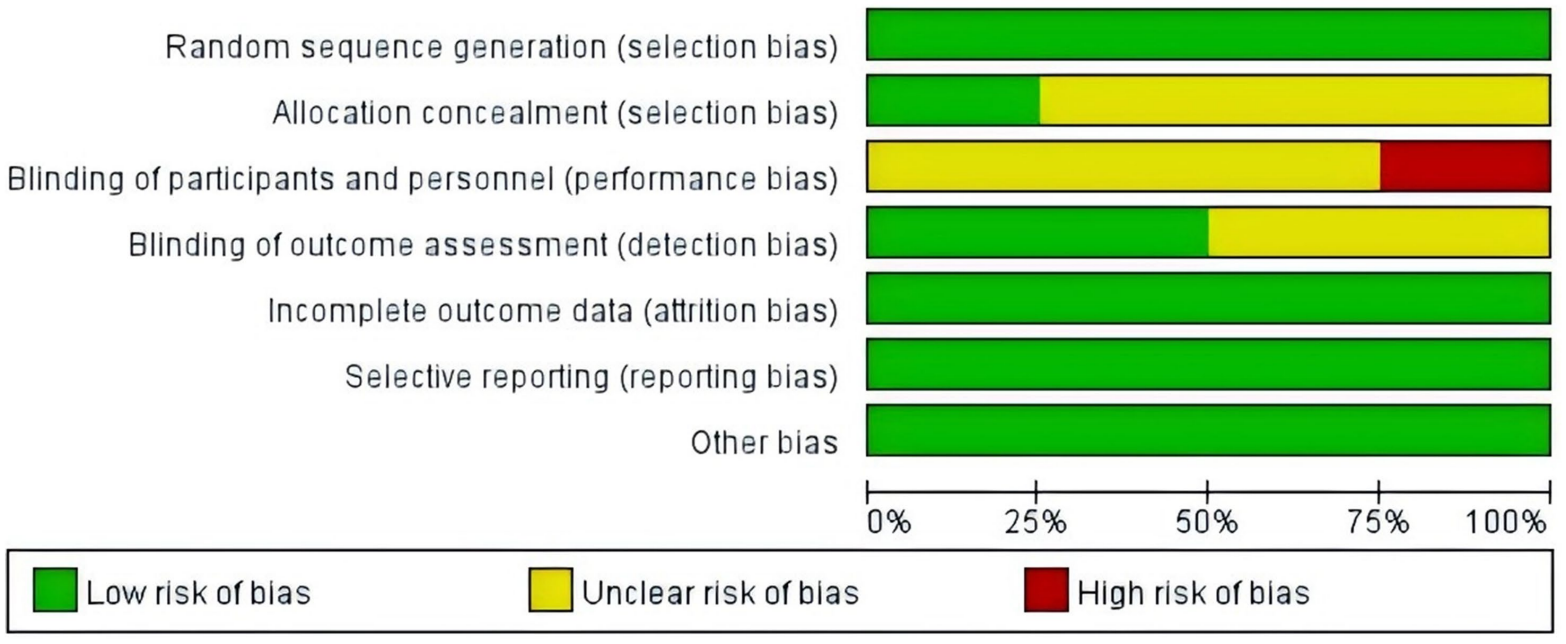
Risk of bias graph.

**Figure 3:**
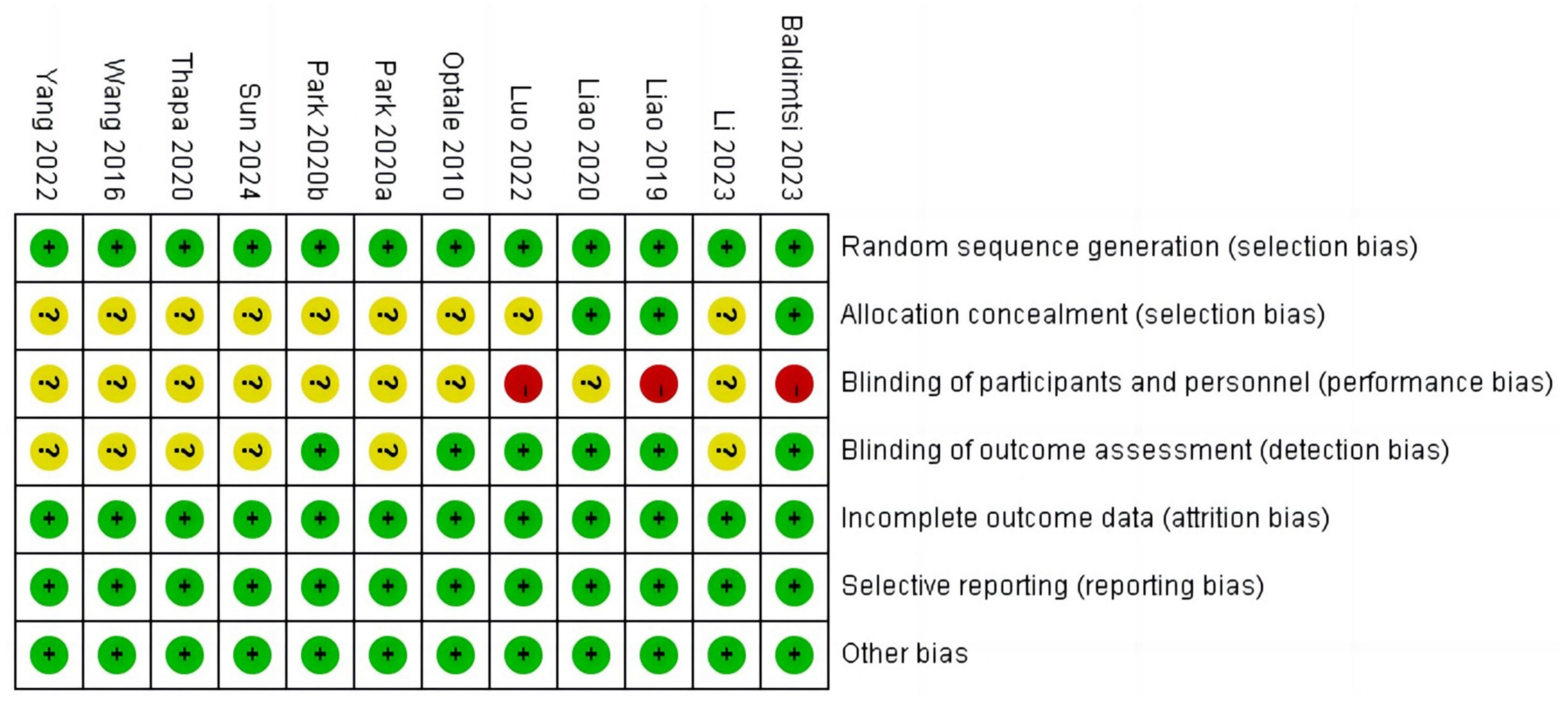
Literature quality and publication bias risk assessment.

Results of the literature quality and publication bias risk assessment are presented in Figures 2, 3. Sensitivity analysis was also performed on the included outcome indicators, and after excluding each literature one by one, there was no effect on the intervention outcome, suggesting no publication bias. Therefore, the results of this study are reliable.

### Publication Bias

**Figure 4:**
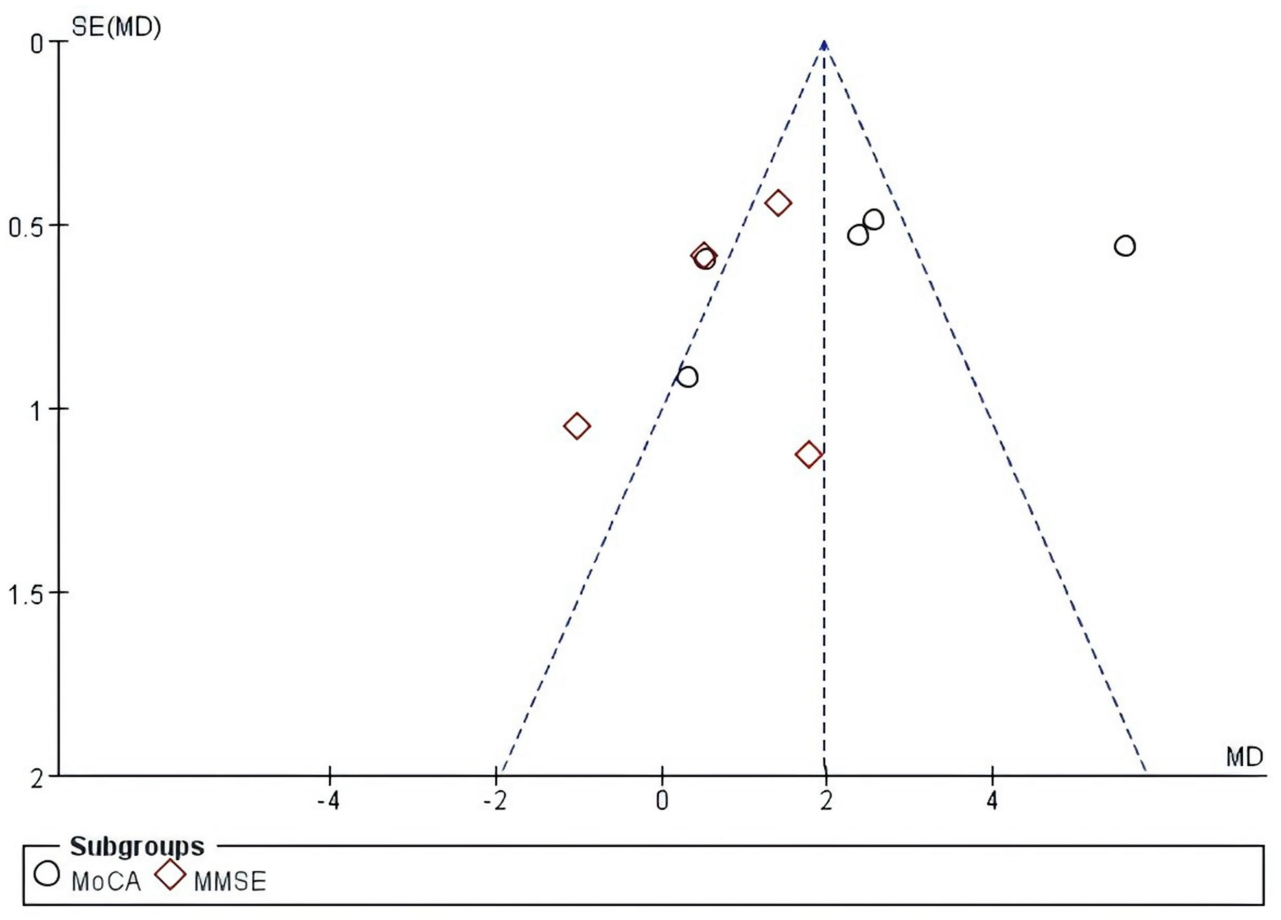
Funnel plot of the global cognitive function.

**Figure 5:**
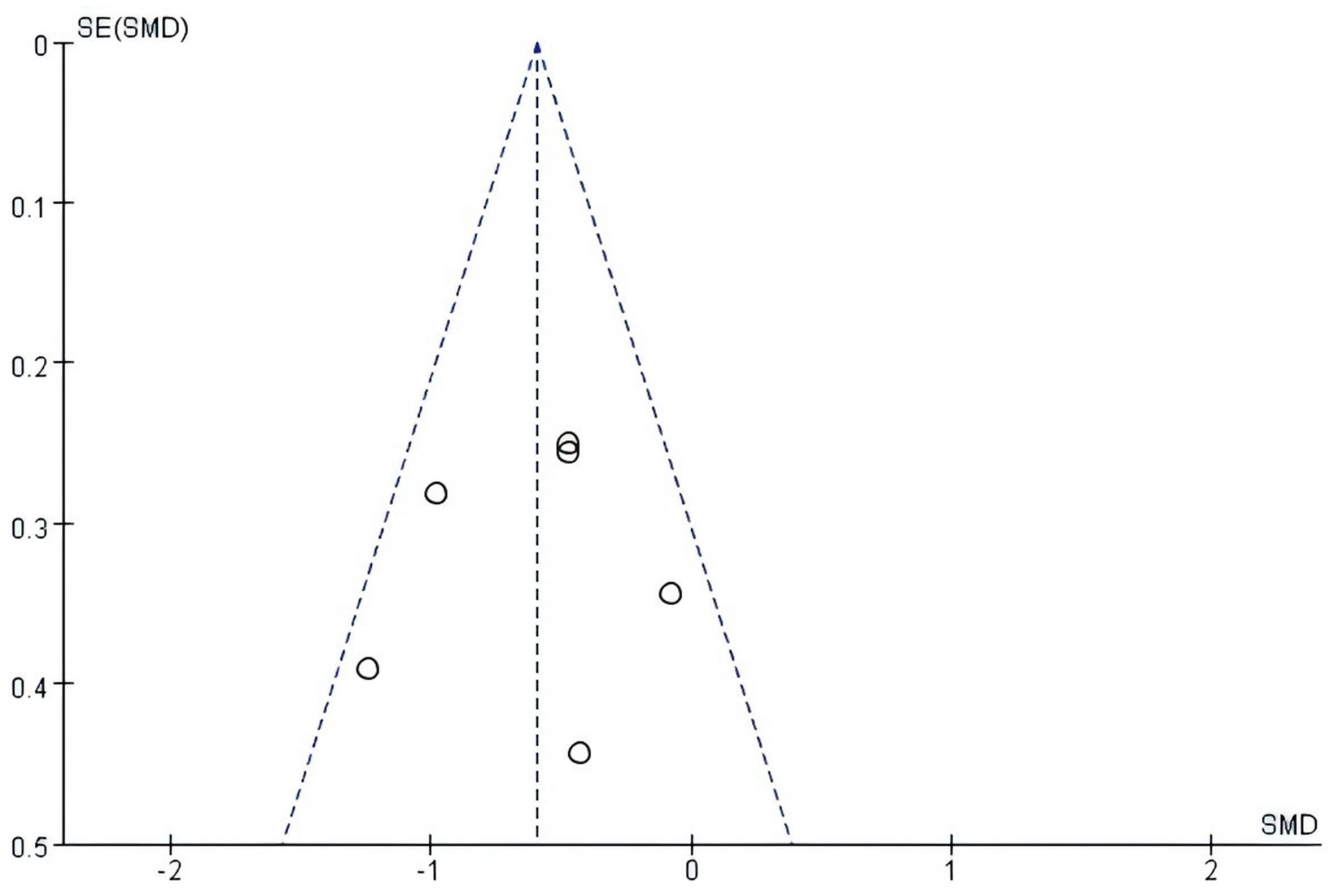
Funnel plot of executive function.

Funnel plots were drawn for the overall cognitive function and executive function evaluation indicators, as shown in Figures 4, 5. It was found that there was a low risk of publication bias due to the symmetry of the left and right scatterplots.

### Main effects and subgroup analysis

#### Global cognitive function

**Figure 6:**
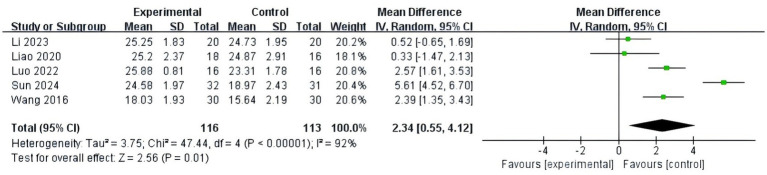
Meta-analysis results of global cognitive function (MoCA).

Five studies (Wang and Lu, 2016; Liao et al., 2020; Sun et al., 2024; Luo et al., 2022; Li, 2023) used the MoCA scale to compare global cognitive function and included 113 MCI patients with high heterogeneity (*p* < 0.01, I^2^ = 92%) among the results. Therefore, a sensitivity analysis was performed using the one-by-one elimination method. After sensitivity analysis, it was found that heterogeneity was not reduced, and a random effects model was used for meta-analysis. The results showed that the MoCA score of the fully immersive VR training was significantly different from the control group (MD = 2.34, 95% CI [0.55, 4.12], *p* = 0.01), as shown in Figure 6.

**Figure 7:**
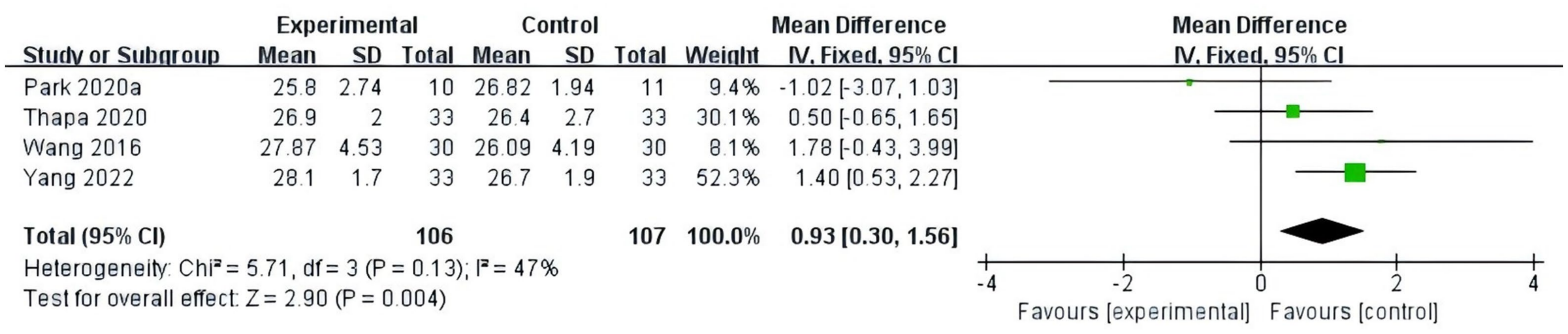
Meta-analysis results of global cognitive function (MMSE).

Four studies (Thapa et al., 2020; Park, 2022; Yang et al., 2022; Wang and Lu, 2016) used the MMSE scale to assess the global cognitive function and included 107 MCI patients with high homogeneity (*p* = 0.13, I^2^ = 47%) among the results. Therefore, a fixed-effects model was used for analysis. The results showed that fully immersive VR training was effective in improving the global cognitive function of MCI patients; the difference was statistically significant (MD = 0.93, 95% CI [0.30, 1.56], *p* < 0.01), as shown in Figure 7.

#### Executive function

**Figure 8:**
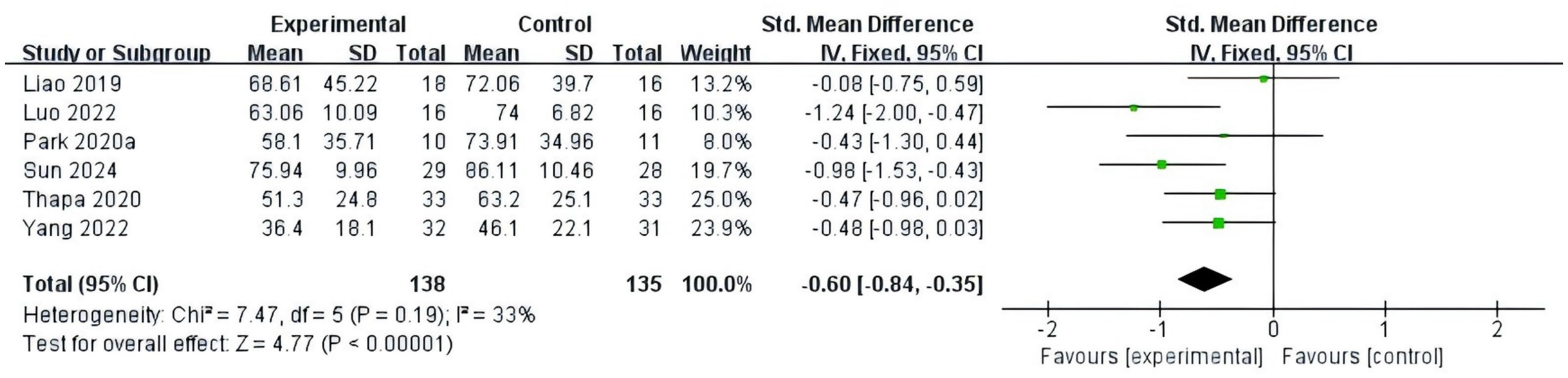
Meta-analysis results of executive function.

A total of six studies (Liao et al., 2019; Park et al., 2020; Thapa et al., 2020; Yang et al., 2022; Sun et al., 2024; Luo et al., 2022) were included, with TMT-A/B or SCWT as the outcome indicator, so SMD was used for analysis. This study included 135 patients with MCI and showed low heterogeneity (*p* = 0.19, I^2^ = 33%). Therefore, a fixed-effects model was used for meta-analysis. The results showed that fully immersive VR training can improve the executive function effectively of MCI patients, and the difference is statistically significant (SMD = -0.60, 95% CI [−0.84, −0.35], *p* < 0.01), as shown in Figure 8. Subgroup analysis was carried out in this study to compare the effects of intervention frequency, intervention period, and total intervention duration, and the results are shown in Table 2.

**Table 2:** Subgroup analysis of the effects of fully immersive virtual reality training on executive functions in patients with mild cognitive impairment.

Subgroup	Number	Group (cases)		Heterogeneity		SMD (95%CI)	Meta-analysis results	
		VR	Control	*I*^2^ (%)	*p*		Z	*p*
Total period of intervention (weeks)								
<10	3	81	80	37	0.20	−0.61 (−0.92, −0.29)	3.73	<0.01
≥10	3	57	55	53	0.12	−0.58 (−0.97, −0.20)	2.98	<0.01
Total frequency of interventions (times)								
<30	4	91	91	10	0.35	−0.58 (−0.88, −0.29)	3.83	<0.01
≥30	2	47	44	76	0.04	−0.55 (−1.44, 0.33)	1.22	0.22
Total duration of intervention (h)								
<40	3	44	43	60	0.08	−0.57(−1.20, 0.13)	1.59	0.11
≥40	3	94	92	14	0.31	−0.62(−0.92, −0.32)	4.11	<0.01

#### Memory

**Figure 9:**
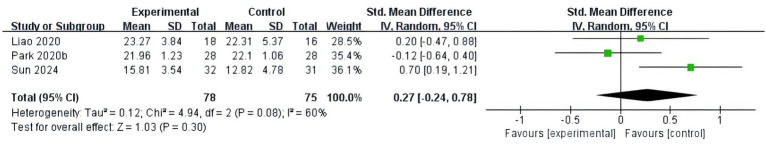
Meta-analysis results of memory.

Three studies (Liao et al., 2020; Park, 2022; Sun et al., 2024) compared memory using VLT/AFT and included 75 MCI patients with high heterogeneity (*p* = 0.08, I^2^ = 60%) among the results. The sensitivity analysis did not reduce heterogeneity. Therefore, a random-effects model was used for meta-analysis. The results did not observe any improvement in memory in MCI patients with fully immersive VR training, and the difference was not statistically significant (SMD = 0.27, 95%CI [−0.24, 0.78], *p* = 0.30), as shown in Figure 9.

#### Attention

**Figure 10:**
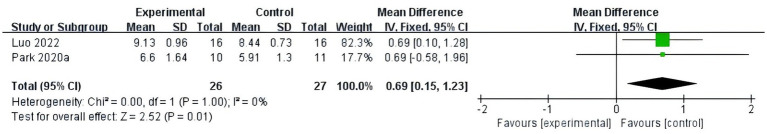
Meta-analysis results of attention.

Two studies (Park et al., 2020; Luo et al., 2022) compared attention using the DST and included 27 MCI patients with high homogeneity (*p* = 1.00, I^2^ = 0%) among the results. Therefore, a fixed-effects model was used for meta-analysis. The results showed that fully immersive VR cognitive training effectively improved attention in MCI patients, and the difference was statistically significant (MD = 0.69, 95% CI [0.15, 1.23], *p* = 0.01), as shown in Figure 10.

#### Daily activity ability

Two studies (Liao et al., 2020; Optale et al., 2010) compared daily activity ability using the ADL scale, but the findings were inconsistent. Optale (Optale et al., 2010) noted that the difference in ADL scale scores between the two groups of patients with MCI was not statistically significant for fully immersive VR training. In contrast, Liao (Liao et al., 2020) noted that fully immersive VR training enhanced the ability of daily living of MCI patients. The ADL scores of the experimental group were significantly higher than those of the control group [control group (18.25 ± 2.04), experimental group (19.77 ± 2.12), *p* < 0.01].

## Discussion

Patients with MCI are often accompanied by a decline in verbal expression, reduced ability to adapt to the environment, and decreased spatial awareness, which can seriously affect their global cognitive function and social adaptability. A significant improvement over control groups was reported in the global cognitive function in fully immersive VR groups, in line with the findings of Wu et al. (2020), Yu et al. (2023), and Yan et al. (2022). The reason may be that fully immersive VR technology provides patients with rich visual, auditory, and tactile stimuli, and the multi-domain task setting enables participants to obtain comprehensive sensory stimulation, which benefits spatial cognition and language function. Analyzed from a neuroanatomical perspective, VR training has been demonstrated to facilitate neuroplastic changes by stimulating brain excitability, promoting synaptic plasticity, and neuronal functional integration in neural networks (Ten Brinke et al., 2019). In addition, based on the cognitive reserve theory, MCI patients can engage in cognitive stimulus-related activities to increase neural reserve to prevent cognitive decline (Vujic et al., 2022). Another study showed that there was no statistically significant improvement in global cognitive functioning with fully immersive VR training, which may be related to the short duration of the intervention and the absence of a dual cognitive and physical training regimen (Park, 2022). Furthermore, some scholars have pointed out that potential confounders such as nutritional status, physical activity, and sleep patterns can also affect study results (Vujic et al., 2022).

Global cognitive functioning in this study was mainly assessed by the MMSE or MoCA or combined effects scales. Several studies have shown that the MoCA scale has higher sensitivity and specificity than the MMSE scale in assessing the cognitive function of MCI patients, which may explain the difference between the MMSE and MoCA scores in the study of Ding (Yan et al., 2021; Scott and Mayo, 2018; Ding, 2023). This study found that both the MMSE and MoCA scores in the experimental group showed significant improvement in cognitive function, which was mainly considered as the fact that compared with semi-immersive or non-immersive VR, fully immersive VR training is more conducive to remodeling cognitive functions by allowing the patients to immerse themselves in virtual environments and extend the training results to real life, as well as realizing the task-oriented and repetitive high-intensity training.

Executive functioning involves a variety of skills such as working memory, inhibitory control, and cognitive flexibility, which are required to cooperate with brain processing to accomplish higher-order tasks. Meta-analysis of this study showed that fully immersive VR training could help to improve the executive function of MCI patients. This may be attributed to the fact that the fully immersive VR technology provided diverse game scenarios and tasks, which further differentiated the training goals of MCI patients and clarified the purposefulness of the rehabilitation. Additionally, the plasticity of the executive function was enhanced, leading to an increase in the patient’s executive function and stabilization of the patient’s clinical status. From a neuroanatomical perspective, virtual reality training scenarios are characterized by high levels of visual realism, which has been shown to enhance the production of brain-derived neurotrophic factor (BDNF) and improve the capacity to store and process information.

Subgroup analyses revealed that a significant improvement in executive function was observed only when the duration of intervention was 40 h or more. Conversely, excessive training frequency (30 times or more) had a detrimental effect on the intervention outcome. The reasons for these findings are twofold: First, executive function involves a range of higher-order cognitive functions, making it more challenging to improve and necessitating complex cognitive training. Therefore, a more extended intervention duration may lead to improved executive function. Second, fully immersive VR training environments possess strong closure, high fidelity, and complete immersion. However, frequent exposure to such an environment for a fixed period may lead to fear, boredom, and resistance in patients with MCI, affecting their motivation and training efficiency. Additionally, another study found no statistically significant improvement in executive function with fully immersive VR training compared to conventional treatment (Park, 2022). This could be attributed to the short intervention time and the need for consideration for intervention frequency and other follow-up data. These findings underscore the importance of ensuring sufficient intervention time and period in future research while also avoiding overly frequent training, which can compromise the effectiveness of the intervention.

Attention is necessary for individuals to allocate cognitive resources and maintain task processing while performing tasks (Yang et al., 2022). Fully immersive VR training improves attention in patients with MCI mainly because patients receive multimodal visual and auditory stimuli as well as complete functional reality tasks in fully immersive virtual scenarios, which significantly enhances the continuous integration of practical perceptual and attentional stimulation aspects. Currently, the strategy of fully immersive virtual reality training focuses on complex modules such as educational games and supermarket shopping, with less emphasis on attention training. It is worth noting that attention is the basis of other cognitive functions. Patients with attention deficits have difficulty focusing on the training process, resulting in poor improvement in cognitive function. Therefore, it is recommended that medical staff appropriately increase attention training sessions to comprehensively promote the recovery of patient’s cognitive function.

Memory is comprised of both short-term and long-term memory processes, and the decline in memory function is most pronounced during the cognitive decline observed in patients with MCI. The limited effect of fully immersive VR training in improving memory remains consistent with the findings of Wu (Wu et al., 2020) and Yu (Yu et al., 2023). The rationale for this conclusion was that only two papers were included in the study, and false-negative results may have occurred due to the limited number of documents. Furthermore, it has been suggested that potential confounding variables, such as nutritional status, physical activity, and sleep patterns, may also influence the intervention effect (Ding, 2023). In the future, the effects of fully immersive virtual training on memory function can be further studied by incorporating more research and increasing the duration of the intervention.

The ability of daily living requires complex cognitive processing, involving working memory, attention, processing speed, and other cognitive domains (Luo et al., 2022) Therefore, this study considers that abilities of daily living must be taken into account when assessing cognitive function. At present, there are fewer original studies on the ability of daily life of MCI patients through fully immersive VR training, and the results need to be further examined. Liao found that fully immersive VR training enhanced the ability of daily life activities of MCI patients, and the reason for this is considered to be the fact that fully immersive VR achieves more precision and completeness in daily life training through multi-module training of memory and executive function (Liao et al., 2020). It is worth mentioning that enhancing daily living ability is a complex integration process. It is not the case that improvement in a single cognitive domain can improve the ability to live life, which explains why there is difference in ADL scale scores between the two groups of MCI patients is not statistically significant in the study by Optale (Optale et al., 2010). However, although the effect of the intervention needs to be further verified, it cannot be ignored that fully immersive VR training brings positive feedback from visual, auditory, and other proprioceptive senses through personalized rehabilitation, ultimately improving patient engagement and motivation.

In conclusion, fully immersive virtual reality training improves global cognitive function, executive function, and attention in patients with MCI. However, several limitations to this study must be addressed. First, the results of this meta-analysis were limited to fully immersive virtual reality training, while the type of study was restricted to RCTs, so future studies with larger sample sizes and higher quality are still needed to validate the above findings further. Second, the quality of the included studies was not high, and the risk of bias in some studies was unclear, so the conclusions may need to be treated with more caution when applied to clinical practice. Third, only Chinese and English literature was included, which may have resulted in the omission of high-quality literature in other languages. Therefore, it is recommended that future studies include more databases and languages for retrieval to improve the reliability of the research.

## Data Availability

The original contributions presented in the study are included in the article/supplementary material, further inquiries can be directed to the corresponding author.
